# The 2025 ISCB Overton Prize Award—Dr James Zou

**DOI:** 10.1093/bioinformatics/btaf261

**Published:** 2025-07-15

**Authors:** Mallory L Wiper

**Affiliations:** The International Society for Computational Biology, Leesburg, Virginia, United States

**Figure:**
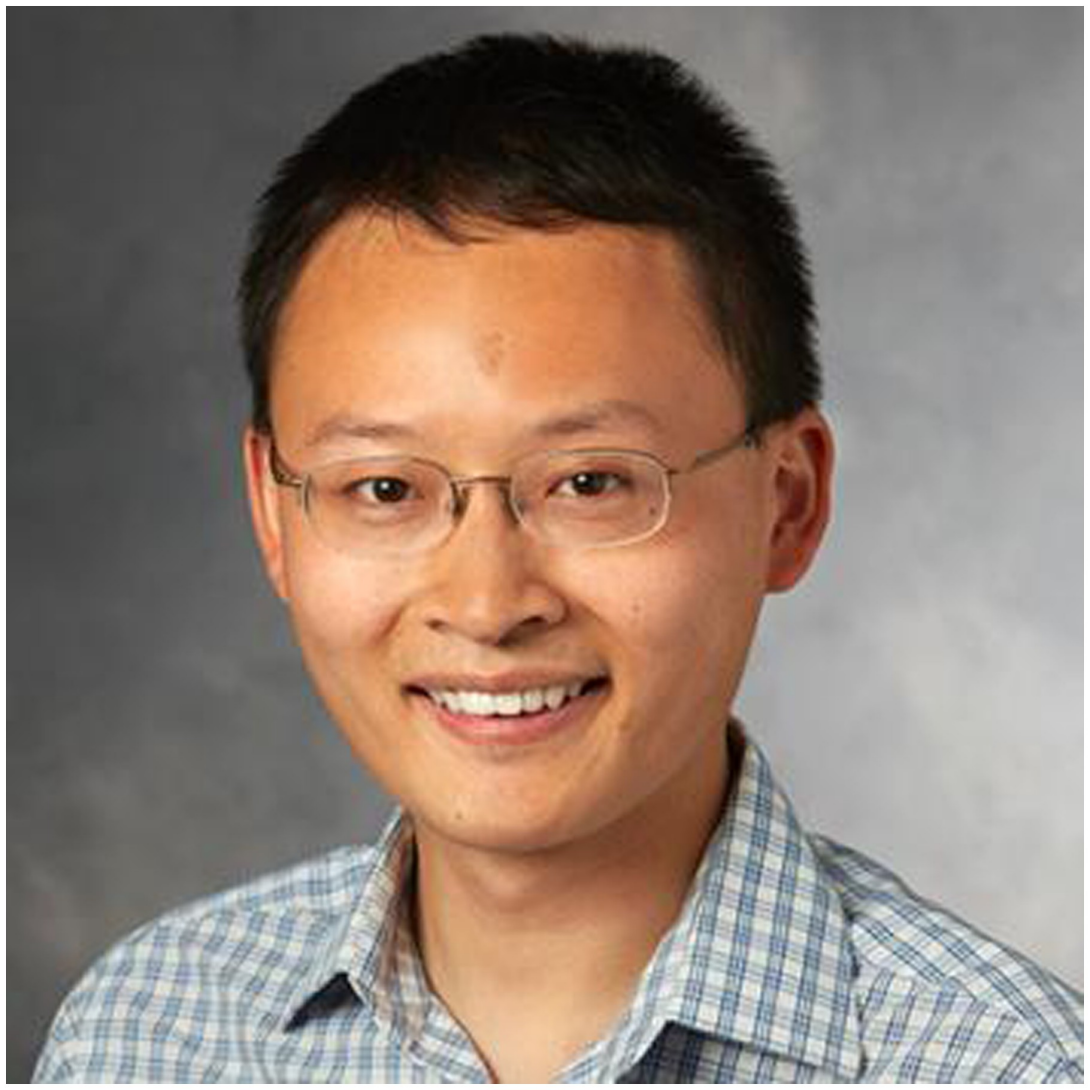


The ISCB Overton Prize is awarded to a scientist for their significant contributions to computational biology. This year, at the 33rd annual conference on Intelligent Systems for Molecular Biology and the 24th European Conference on Computational Biology, the International Society for Computational Biology has the pleasure of honoring Dr James Zou with this award!

## A spark for science and mathematics

Long before his career in computational biology, Dr James Zou was drawn to the challenge and logic of mathematics. This fascination began in elementary school, when he took part in math competitions and discovered that solving math puzzles was like a game—challenging, but fun! These early experiences with logical problem-solving laid the foundation for a lasting interest in mathematics.

During middle school, a classic science fiction series gave Zou’s interest in math a new direction. When Zou read Asimov’s *Foundation Trilogy*, he was captivated by the premise that a mathematician could model the behavior of entire societies and predict the rise and fall of civilizations. The idea that math could be so influential sparked a growing interest in science and showed him the power of applying mathematical models to real-world problems.

## Discovering computational biology

The love of math and science accompanied Zou into his undergraduate years in university. Because of this interest, he took part in a semester abroad program which brought him to Budapest, Hungary. It was in a course he took while in Budapest that Zou was first introduced to computational biology, where he studied the textbook *The Biological Sequence Analysis* by Durbin and colleagues. The focused study on such an influential book helped shape Zou’s understanding of computational biology and bioinformatics, especially with how computational models could be applied to DNA sequence analysis.

Around 2010, Zou began studying for his PhD at Harvard, where he focused on computer science, algorithms, and machine learning. However, after reading about the groundbreaking discovery of Yamanaka factors—transcription factors capable of reprogramming mature cells into a pluripotent state—Zou’s research interests expanded to include biology. The idea that cells could be reprogrammed to an earlier state astonished Zou, prompting him to ask, “How is this even possible?” This curiosity led him to explore the use of computation and machine learning in uncovering the mechanisms behind Yamanaka factors and improve the efficiency of reprogramming experiments.

## Guidance and growth through mentorship

Zou considers himself fortunate to have learned from many mentors throughout his career, crediting three groups of people who have significantly shaped his growth as a researcher.

First were his early academic advisors, two of which stood out for their lasting influence: Dr David Parkes, a computer scientist and AI researcher, and Dr Brad Bernstein, a biologist specializing in epigenomics. Zou admired Parkes’s ability to think deeply about complex questions and identify problems whose solutions would have the greatest societal impact. Zou says that Parkes was someone from whom he learned a great deal and whose example he strives to follow in his own career.

From Bernstein, Zou was introduced to the biological mechanisms behind cellular reprogramming and epigenomic regulation. Bernstein’s encouragement to engage in experimental work broadened Zou’s scientific perspective, strengthening his approach as a computational biologist.

As he advanced in his career, Zou also learned a great deal from senior collaborators, especially during his early faculty years at Stanford. Among them, Dr Howard Chang—now Chief Scientific Officer at Amgen—Dr Tom Montine, and Dr Anne Brunet were particularly influential. Co-advising students alongside Chang, Montine, and Brunet allowed Zou to bridge his computational expertise with their wet lab research. These collaborations enriched his perspective of how computational tools could directly impact biological discovery and shaped the way he approached interdisciplinary work.

Zou also credits his own students as important mentors. He sees them not only as trainees but as vital collaborators who bring fresh ideas and challenge him to explore new directions. One such student was Dr Ruishan Liu, whose research integrated genomic data with electronic health records to predict patient responses to cancer treatments. Collaborating with Liu, and many others, has been a continual source of learning for Zou, pushing his lab’s research into unexpected places.

## Guiding students toward discovery

With all the lessons he’s learned from his own mentors and throughout his career, Zou likens his mentoring philosophy to a camping trip: It’s the job of the camp leader to find the most suitable place to camp and provide the tools needed to set up a good campsite, but when it comes to exploration of the surrounding area, the camp leader should let the *campers* take the lead. That is to say: As a mentor, a principal investigator (PI) should identify the most promising topics of research with interesting questions to explore. They should make sure their students are well-equipped to explore those questions and navigate the research landscape, stepping in to help clear obstacles when necessary. Most importantly, though, the PI should encourage their students to take the lead and explore ambitious questions beyond their immediate research area if they have a new idea or unanswered question they want to explore.

In his lab, Zou has a system where any potential new PhD student he takes into his research group does a short-term “rotation” where they work directly with him on a mini project for two to three months. This process helps both Zou and the student determine whether their research styles, tastes, and interests are compatible before a long-term commitment to a PhD project. Zou says that this focused and deliberate process of bringing students into his lab has enabled him to recruit students from diverse research backgrounds and interests, including computer science, statistics, and medicine.

From this position of PI and mentor, Zou says he’s become more focused on the broader societal impact of research, considering how the research and methods developed in his lab can directly contribute to bigger questions. Though he still researches foundational biological questions, considering the broader impacts of research has led him to prioritize projects with translational applications.

## Unexpected discoveries and future directions

Reflecting on his research, Zou mentioned a surprising discovery in 2020 when exploring whether tissue images could be used to predict gene expression in different tissue neighborhoods. A PhD student in his lab was keen to explore this question more closely and ultimately found that there were hundreds of genes whose expressions could be accurately predicted from morphology of tissues as seen in simple tissue images. This breakthrough in spatial biology demonstrated that imaging data could be computationally mapped to gene expression profiles. What’s more, this finding led to further research in Zou’s lab on spatial motifs—the way that cells arrange themselves in local patterns in tissues—which are predictive of how patients respond to immunotherapies and different cancer treatments.

These findings have led to a continued fascination and excitement about mapping cellular environments across tissues and how integrating data from imaging, genomics, and proteomics will lead to a better understanding of human biology.

In addition to spatial biology, Zou is currently interested in developing AI scientist agents who can help make biomedical discoveries. Specifically, he’s developing virtual labs where AI “professors” and AI “students” work together to solve different scientific problems, like designing new molecules and proteins and analyzing different computational biology tasks. In fact, these AI lab systems have already been applied to designing antibodies for COVID variants, with experimental validations showing very promising outcomes, demonstrating real-world potential!

## Reflections on receiving the Overton Prize

When asked how he felt about being named the ISCB 2025 Overton Prize award winner, Zou said that he’s “incredibly grateful, honored, and humbled” to be the recipient of the award and that it’s “quite surreal” to be following in the footsteps of so many of his scientific heroes who have previously won the Overton Prize! He also emphasized that the award isn’t just a personal achievement but is a recognition of the students, collaborators, and mentors he’s worked with throughout his career who’ve made every day of doing research inspiring and fun.

